# Integrating Gut-Brain Axis: Exploring the Neurogastrointestinal Interactions and Therapeutic Potentials in Autism Spectrum Disorder

**DOI:** 10.2174/0118715303369166250325110016

**Published:** 2025-04-07

**Authors:** Manjeet Kumar, Sidharth Mehan, Tarun Sharma, Aakash Kumar, Zuber Khan, Arun Kumar Sharma, Ghanshyam Das Gupta, Nitish Kumar

**Affiliations:** 1 Division of Neuroscience, Department of Pharmacology, ISF College of Pharmacy, Moga, Punjab, India (Affiliated to IK Gujral Punjab Technical University), Jalandhar, Punjab, 144603, India;; 2 SRM Modinagar College of Pharmacy, SRM Institute of Science and Technology (Deemed to be University), Delhi-NCR Campus, Modinagar, Ghaziabad, Uttar Pradesh, 201204, India;; 3 Department of Pharmaceutics, ISF College of Pharmacy, Moga, Punjab, India (Affiliated to IK Gujral Punjab Technical University), Jalandhar, Punjab, 144603, India

**Keywords:** Gut-brain axis, autism spectrum disorder, gastrointestinal symptoms, microbiota, neurotransmitters, microbiome-based therapies

## Abstract

This comprehensive review critically examines the gut-brain axis (GBA) and its implications in autism spectrum disorder (ASD). The GBA is a complex, bidirectional communication network that integrates the gastrointestinal tract, the central nervous system, and the gut microbiota. This axis is mediated through various physiological pathways, including the enteric nervous system (ENS), the vagus nerve, immune responses, and metabolic activities of gut microorganisms. ASD, a developmental disorder marked by social impairments and repetitive behaviors, presents with notable neurological irregularities. The review highlights the increased prevalence of gastrointestinal (GI) disturbances in individuals with ASD, suggesting a potential link between GI symptoms and the severity of ASD-related behaviors. This correlation is supported by evidence of altered gut microbiota composition in ASD, indicating significant interactions between the gut environment and neurological health. Moreover, the pathophysiology of ASD is explored with an emphasis on genetic and environmental contributions to neurodevelopmental impairments. Key topics include synaptic dysfunction, the roles of neurotransmitters like GABA and serotonin, and the impact of gut-brain interactions on ASD progression. Specifically, this review addresses how gut microbiota may influence metabolic alterations, immune dysregulation, oxidative stress, mitochondrial function, and neurotransmitter production in ASD. Emerging research on microbiome-based therapies for ASD is discussed, focusing on the potential of probiotics, prebiotics, and faecal microbiota transplantation (FMT) as novel interventions. Ethical considerations in this burgeoning field are also considered, highlighting the necessity for rigorous scientific inquiry and ethical oversight. The review advocates for a multidisciplinary approach to understanding and addressing the complexities of ASD. By integrating insights from genetics, neuroscience, psychology, and gastroenterology, a more comprehensive understanding of the role of GBA in ASD can be achieved. This interdisciplinary perspective is crucial for developing effective, individualized treatments and improving the quality of life for individuals with ASD.

## 1 INTRODUCTION 

### 1.1 A Brief Overview of the Gut-brain Axis and its Significance

The bidirectional physiological connection that facilitates information exchange between the microbiota, the intestines, and the brain is referred to as the gut-brain axis (GBA) [1]. This axis comprises signalling mechanisms between the gastrointestinal tract and the central nervous system [2]. The brain and gastrointestinal tract are connected through various pathways, including the vagus nerve, enteric nervous system (ENS), immune system, and gut microorganism metabolic processes. The ENS governs local regulation, while the prevertebral ganglia receive impulses from the ENS and CNS. By connecting motor and sensory neurons, the ENS executes functions comparable to those of the brain and spinal cord. The CNS controls the gastrointestinal system through autonomic nervous and neuroendocrine systems, affecting smooth muscle, organs, and blood vessels. The fourth level of brain centers is where information from the cortex and subcortical regions converges to specific brain stem nuclei and the basal ganglia [3]. This review critically examines the GBA in ASD, highlighting the role of altered gut microbiota in neurological and behavioral symptoms and exploring emerging microbiome-based therapies as potential interventions.

#### 1.1.1 Literature Search Strategy

A comprehensive literature search was conducted using the following electronic databases: PubMed, Scopus, and Web of Science. The search strategy included a combination of keywords, including Gut-Brain Axis (GBA), Autism Spectrum Disorder (ASD), Gastrointestinal (GI) symptoms, microbiota, neurotransmitters, and microbiome-based therapies. For this review, studies were included if they were research papers. Studies were excluded if they had inadequate methodology, were not relevant to our research question, were duplicate publications, and were non-peer-reviewed articles.

To ensure the most comprehensive and reliable analysis, we employed a systematic literature search with clearly defined inclusion and exclusion criteria, prioritizing studies with rigorous methodology and high-quality data.

### 1.2 Introduction to Autism Spectrum Disorder (ASD)

Autism spectrum disorder (ASD) is a continuous developmental condition distinguished by repetitive, limited social challenges, and restricted behaviour [4]. Social behaviours are characterised by difficulties with maintaining eye contact, emotional regulation, comprehending the emotions of others, and engaging in a diverse array of activities and interests [5]. Detailed indications and symptoms of ASD can be found in the Diagnostic and Statistical Manual of Mental Disorders-V [6]. ASD involves neurological abnormalities in brain regions, including the neocortex, amygdala, hippocampus, and basal ganglia. In 2014, the prevalence of ASD among 8-year-old children was 1.7%, or 1 in 59. According to the surveillance statistics from the CDC, males were four times more likely to be affected than females [6]. Approximately 6% of cases exhibiting autistic traits have identified aetiologies, such as neurofibromatosis, tuberous sclerosis, or fragile-X syndrome [7]. A correlation between GI abnormalities and the severity of behavioural problems associated with ASD suggests that gut-brain interaction plays a role in the pathogenesis or progression of ASD [8]. An elevated concentration of blood serotonin (5-HT) is detected in a subset of children diagnosed with autism [9].

ASD is characterised by the association of several genetic mutations with proteins that perform synaptic functions, including SHANK, CNTNAP, NLGN, and NRXN. The UBE3A genes, along with the chromosomal loci 15q11-q13 and 16p11.2, are CNVs associated with ASD. Single-gene mutations linked to ASD can also induce other neurodevelopmental disorders like Rett syndrome, Fragile X syndrome, and tuberous sclerosis [10]. Specific characteristics of Fragile-X syndrome include impaired or nonexistent fragile X mental retardation protein (FMRP) expression, as well as aberrant neuronal migration in the hippocampus and cerebellum [11, 12]. In a study, the administration of IgG from mothers of children with ASD to rhesus macaques led to hyperactivity and stereotypical behaviour in the young, which closely resembled symptoms associated with ASD [13]. ASD, a multifactorial disorder, is characterized by lifelong variation in development patterns in diverse white-matter regions influenced by oligodendroglial cells, providing essential neurotransmission and axonal conduction factors [14]. Environmental and genetic factors can contribute to neurodevelopmental impairment, which can induce microstructural abnormalities, including axon diameter and cell density alterations. Moreover, the integrity of white matter, grey matter, the amygdala, hippocampus, and prefrontal cortex may be compromised by macrostructural defects, which may result in electrophysiological disruptions. ASD may be exacerbated by the socioemotional task-based alterations that can be indicative of these abnormalities [15]. Due to speculative limitations in existing studies, conditions, such as gastrointestinal immune/inflammation-mediated pathology in ASD and 'leaky gut' syndrome, characterised by increased intestinal permeability, have not been conclusively identified [16]. ASD animal models, in addition to postmortem and neuroimaging investigations, uncover irregularities in several brain regions, including the amygdaloid nucleus, frontal cortex, cerebellum, hippocampus, and cerebello-thalamo-cortical pathways [17].

Recent findings suggest that glial cells may play a significant role in the neuroanatomical and behavioral alterations linked to ASD. Oligodendrocytes and astrocytes can induce neurochemical imbalances in the autistic brain, potentially causing neurotransmission or axonal conduction modifications. Genetic depletion of glutamate transporter-1 (GLT-1) in astrocytes enhances excitatory neurotransmission, resulting in repetitive behaviours similar to ASD in mouse animal models [18]. On the contrary, phosphatase and tensin homolog (PTEN) mislocalization results in premature myelination and oligodendrocyte maturation. Brain-derived neurotrophic factor (BDNF) is essential for the formation of synapses, survival, growth, and differentiation of neurons [19]. This implies that autistic individuals may encounter neuronal communication failures and challenges with social communication and language impairments. Interneuronal communication depends on various mechanisms, including structural synaptic foundations, activity-dependent protein synthesis, gene silencing, and expression upregulation [20]. Primary neurotransmitter gamma-aminobutyric acid (GABA) inhibits glutamate decarboxylation *via* glutamate decarboxylase. N-methyl-D-aspartate receptor dysfunction in GABAergic neurons can result in neurotoxicity, excessive glutamate release, and postsynaptic glutamate neuron disinhibition [21]. Abnormal GABA levels are frequently observed in children with ASD with neurological injury; these abnormalities could play a substantial role in the pathophysiology of the disease (Fig. **1**). [22, 23].

### 1.3 Gut Microbiota and its Impact on ASD

Several factors have been linked to the influence of the gastrointestinal microbiota on metabolic changes in individuals with ASD, including aberrations in neural function, oxidative stress, mitochondrial function, and immune system functionality. These effects include altered neurotransmitter synthesis, microglia activation, increased blood-brain barrier (BBB) permeability, and synaptic dysfunction [24]. By interacting with commensal bacteria, probiotics, also known as live bacteria or psychobiotics, have been shown to enhance mental health. These interactions may alter the gut-brain axis, which may be advantageous for neuropsychiatric disorders [25-27].

The brain affects gastrointestinal microbiota composition through the hypothalamus-pituitary-adrenal (HPA) axis, which can elevate proinflammatory cytokines and cortisol levels in the blood. This is facilitated by adrenocortical and pituitary glands, as well as stimulation of hypothalamic neurons with arginine/vasopressin and corticotropin-releasing hormone (CRH). The cytokine signal can traverse the BBB through vagal afferent stimulation. Plasma cortisol, produced as the end product of peripheral cytokine-stimulated HPA axis activation, acts as a negative feedback mediator [28]. Furthermore, it has been reported that probiotics and prebiotics can significantly improve gastrointestinal and autism symptoms. This supplement promotes the growth of helpful bacteria (*Bifidobacterium* and *B. longum*) while inhibiting the development of harmful bacteria [29, 30].

The heterogeneity of ASD significantly impacts the potential effectiveness and modification of microbiome-based therapies. Microbiome composition and gut-brain axis interactions are known to differ between individuals with ASD, potentially influenced by factors, such as age, sex, diet, genetic predisposition, and co-occurring medical conditions (*e.g.,* gastrointestinal disorders, immune dysregulation). Consequently, the therapeutic effects of probiotics or other microbiome-based interventions may not be uniform across all ASD subtypes [31]. For instance, individuals with autism who exhibit prominent gastrointestinal issues might respond more robustly to interventions targeting gut health, whereas others characterized by severe neuroinflammation might require a different therapeutic approach. These gut-microbiome-focused therapies provide a viable strategy for reducing mental health issues [32]. Maternal factors, including toxicant exposure, infection, autoimmunity, and the presence of anti-fetal brain autoantibodies, can impact neurodevelopment during critical periods [33]. These factors can also increase an individual's susceptibility to cytokine dysregulation. ASD progression may be influenced by microbial modulation through the use of pro/pre/synbiotics/FMT *via* direct neural pathways, endocrine, and immunological pathways mediated by the gastrointestinal microbiota. These metabolic pathways decrease systemic and intestinal inflammation.

Furthermore, through their regulation of neuroplasticity, epigenetics, and gene expression, neuroactive metabolites, such as short-chain fatty acids (SCFAs), can impact the central nervous system (CNS) [34]. Particular microorganisms can stimulate the vagus nerve, a key conduit for communication between the brain and gut. For example, treatment with *L. reuteri* stimulates the vagus nerve, increasing oxytocin levels in the brain and improving the behavioral aspects of brain function [35]. The microbiota produces neurotransmitter precursors, such as glutamate, dopamine, tryptophan, γ-aminobutyric acid (GABA), serotonin (5-hydroxytryptamine), and tryptophan. Suppressing inhibitory neurotransmission with probiotics (*e.g.,* increasing GABA concentrations) may assist in restoring the equilibrium between excitatory and inhibitory signals and ameliorating social interaction deficits associated with ASD [36].

### 1.4 Clinical Studies on ASD

Clinical studies on ASD explore its neurodevelopmental basis, genetic factors, and therapeutic interventions. In a double-blind crossover study, intravenous oxytocin treatment enhanced social learning in ASD patients as compared to a placebo [37]. According to another study, patients with ASD exhibited much less repetitive behaviour after receiving oxytocin as opposed to a placebo [38]. Additionally, a 6-week, multicenter, randomised, double-blind, placebo-controlled, fixed-dose research study that included clinical and investigative settings was conducted in the United States from 2007 to 2010. This study involved 16 locations consolidated into nine centers for analysis, followed by three weeks of screening, six weeks of randomized double-blinding, and six months of open-label extension [39].

Second-generation antipsychotics raise severe concerns regarding the long-term safety and tolerability of autism sufferers. To reduce the risk of treatment-related adverse events, such as sedation and tardive dyskinesia, as well as metabolic adverse events (weight gain, dyslipidemia, and hyperglycemia), it is crucial to establish the lowest effective dose for these medications because their long-term use is required. When risperidone was first used to treat autism, the earliest confirmatory trials were unable to determine the lowest effective dose [2, 40].

### 1.5 Importance of Investigating the Gut-brain Connection in Autism

The gut-brain axis maintains physiological homeostasis through bidirectional, tightly regulated communication between the ENS and the brain [41]. Since the initial suggestion of a connection between autism and gut microbiota in the early 20^th^ century, an increasing body of research has underscored the significance of interactions between the gut, brain, and microbiome in the pathogenesis and manifestation of ASD [42]. Investigating the relationship between the brain, gut, and microbiota in ASD is a particularly interesting area of study, especially in juvenile populations [31]. Extensive research is devoted to the gut microbiome, which comprises the collective genome of an estimated 100 trillion microorganisms in the gastrointestinal tract [43].

Bidirectional signalling *via* the vagal nerve, dysregulation of metabolic pathways involved in the production of neurotransmitters (*e.g.,* serotonin and Gamma-aminobutyric acid), and direct effects on the release of gut peptides with modulatory functions from enteroendocrine cells are all key mechanisms of the gut-brain interaction [44]. Pediatricians treating children with ASD are increasingly recognizing the potential link between the disorder and gastrointestinal issues [45]. According to the research, the microbiota-gut-brain axis has been implicated in neurodevelopmental disorders, such as attention deficit hyperactivity disorder (ADHD), ASD, and Rett Syndrome [46]. GI abnormalities observed in individuals with autism are associated with a greater brain mass, probably attributable to an increased density of neurons in the prefrontal cortex [47].

The gut-brain axis is a bidirectional pathway linking the intestines and the brain, often involving the microbiota [48]. It includes both efferent and afferent signals, which encompass the enteroendocrine system, cytokines, metabolites, digestive products, and neuroactive molecules traveling from the gastrointestinal tract to the brain. On the contrary, efferent signals proliferate from the intestinal wall and include neuroendocrine and autonomic regulation [49]. Maternal immune system models suggest an association between inflammation, gut, and brain dysfunction, and microbiome changes affecting gut and brain function. This is because IL-6 controls the microbiome and damages mitochondria. The regulation of IL-6 and the microbiome are mutually influential. In a study, gut microorganisms, including *Clostridium tetani*, *Desulfovibrio spp*., and *Bacteroides vulgatus*, were reported in 46-84% of individuals with ASD [50]. Animal models demonstrated that the intestinal microbiome influences social behaviour bidirectionally [51]. Adaptive immune cells and microbe-sensing antigen processing cells (APCs) stimulated at the intestinal site are transported to distal tissue sites *via* systemic diffusion [52]. Clinically, ASD patients have a reduced abundance of anti-inflammatory and SCFA synthesizing species, including *Faecalibacterium* species, compared to the control group [53]. According to recent studies that analyzed the human genome, two gene clusters were identified in the brains of individuals with psychiatric disorders. One of these clusters was linked to increased inflammation, while the other was associated with decreased mitochondrial function [54].

Analyses of peripheral blood samples revealed significant changes in the expression of immune cells, which may account for improvements in GI function [55]. Memory and learning processes are influenced by the microbiome of the brain, which modulates the synthesis of neurotransmitters, including serotonin, GABA, and BDNF. These neurotransmitters influence various bodily functions by interacting with receptors on enteroendocrine cells (Fig. **2**) [56-58].

## 2 THE MICROBIOME: UNDERSTANDING THE BASICS

### 2.1 Role of Gut Microbiome in Health Maintenance and its Composition

“Microbiota” denotes the collection of microorganisms that reside within a particular habitat. These communities are distributed across various anatomical surfaces, such as the oral cavity, genitourinary tract, gastrointestinal tract, and epidermis. Genetic material is present in each bacterium [59]. “Microbiome” refers to the entire set of genes and genomes comprising the microbiota [60]. Currently, DNA sequencing techniques that are both efficient and economical enable the determination of the microbiome's distinct composition. The composition of the gut microbiome is influenced by various environmental factors, including but not limited to age, gender, mode of birth, lactation, medication usage, hygiene practices, and exposure to toxins [61-64].

Microbially mediated therapies can substantially improve the quality of life for patients diagnosed with ASD. Increasing evidence implicates the gut-microbiota-brain axis in the development of this disorder. However, dietary factors significantly impact the composition of the intestinal microbiome; therefore, dietary interventions for specific subsets of ASD could be considered a form of microbially mediated therapy. A more profound comprehension of the influence of nutrition on human health and behaviour is imminent. Despite the lack of significant evidence from small, meticulously conducted trials, gluten-free and casein-free (GFCF) diets are widely implemented among individuals with ASD [65, 66].

### 2.2 Impact of Diet on Gut Microbiome Composition and Diversity

It was initially reported in 1977 that dietary protein affects the gut microbiome. A study reported that people who consumed a high-beef diet had lower levels of *Bifidobacterium adolescentis* and higher levels of *Bacteroides* and *Clostridia* than those who consumed a meatless diet [67]. It has been observed, for instance, that whey and pea protein extract ingestion increases gut-commensal *Bifidobacterium* and *Lactobacillus* and reduces pathogenic *Bacteroides fragilis* and *Clostridium perfringens* [68].

The sequencing data identified associations between the microbiota and 126 different environmental factors, including diet, disease, and medication use. Higher intakes of total carbohydrates were found to be most strongly associated with decreased microbiome diversity: Bifidobacteria increased while *Lactobacillus, Streptococcus,* and *Roseburia* genera decreased. The Shannon diversity index decreased with increasing intake levels of total carbohydrates, followed by sugar-sweetened beverages, bread, beer, savory snacks, and, to a lesser extent, consuming total fats, pulses, and legumes. Diversity was also reduced in individuals self-reporting IBS, and antibiotic use was associated with decreases in two species of *Bifidobacterium*. On the other hand, microbial diversity increased with fruit, coffee, vegetable, and red wine intake and, to a smaller extent, eating breakfast and drinking tea. Red wine consumption was found to be associated with an increased abundance of *F. prausnitzii* [69] (Fig. **2**).

### 2.3 Diet-based Modulation

Except for probiotics and prebiotics, neural activity in the CNS is influenced by diet [70]. Microorganisms ferment dietary fibre anaerobically to produce short-chain fatty acids (SCFAs) [71]. SCFA levels denote the quantity of soluble fibre and intestinal flora in the diet [72]. The SCFA distribution, which signifies the equilibrium of advantageous SCFAs (n-butyrate, propionate, and acetate), indirectly assesses the balance of anaerobic organisms in the colon. SCFAs are vital in maintaining intestinal health by providing energy to cells and the body. A diet deficient in soluble fibre, an imbalance in the normal flora of the colon, or an extended intestinal transit time may all contribute to decreased levels of intestinal flora [73].

### 2.4 Novel Technologies in the Gut Microbiota-brain Axis

Innovative methods are needed to adequately examine the complicated mechanisms of the gut microbiota brain axis. Functional magnetic resonance imaging (fMRI) has demonstrated considerable potential as an instrument for investigating gut-brain signalling in humans. An instance of a noteworthy fat-emotion interaction effect on the blood oxygen level-dependent (bold) signal was identified in multiple brain regions using fMRI [74]. This suggests that infusion of fatty acids may reduce neural and behavioural responses to sadness in humans. PhMRI investigations have demonstrated that a bolus of ghrelin diminishes the brainstem-ocular-sensory domain (bold) signal in feeding-activated regions of the central nervous system (CNS) [75]. Conversely, intragastric dodecanoate induces robust activation in CNS regions, including the hypothalamus, limbic areas, and brainstem [76].

### 2.5 Probiotics and Gut-brain Axis in ASD

In children with ASD aged five to nine, probiotic supplementation for three months improved gastrointestinal microbiota, gastrointestinal symptoms, and the severity of ASD behaviours, symptoms, and functioning [77]. The potential correlation between diminished concentrations of specific metabolites and improved eye contact and constipation in children with autism has been suggested [78]. Additionally, the maternal gastrointestinal microbiome significantly influences the incidence of ASD in progeny. Maternal gut flora stimulates Th17 immune cells to combat viral infections, leading to IL-17a secretion and behavioral abnormalities in offspring. Probiotics have shown potential as anti-inflammatory agents in certain chronic inflammatory diseases [79]. *Bifidobacterium infantis* and other beneficial intestinal microbiota convert tryptophan to serotonin, which controls behaviour and mood [80]. Probiotics might alleviate ASD symptoms *via* the modification of neuroactive compounds. Growing evidence suggests genetic and environmental risk factors disrupt the equilibrium between glutamate (Glu)-mediated excitatory and γ-GABA-mediated inhibitory neurotransmission in autism [81, 82].

Psychobiotics play a crucial role in maintaining gut-brain communication, thereby regulating neurotransmitters and stress. They stimulate the synthesis of hormones and neurotransmitters, including noradrenaline (NA), gamma-aminobutyric acid (GABA), and serotonin (5-HT), in the gastrointestinal tract, which may influence neurotransmission in the proximal synapses of the ENS. Stress also affects barrier functionality, leading to the proliferation of pro-inflammatory cytokines and glucocorticoids, which compromise the integrity of the BBB and allow harmful substances to enter. Psychobiotics restore the intestinal barrier's integrity, decrease pro-inflammatory cytokines and glucocorticoids, and elevate anti-inflammatory mediators, improving the blood-brain and gastrointestinal barrier's integrity and reducing inflammation. Central lymphatic vessels also play a role in these effects [82]. It has been demonstrated that long-term administration of *Lactobacillus rhamnosus* to rodents reduces depressive and anxious behaviours by inducing a region-dependent alteration in GABA expression in the brain. Nevertheless, the absence of these neurochemical and behavioural effects in vagotomized mice suggests that the vagus nerve plays a crucial function in communication between the gut microbiota and the brain [83, 84]. Using diverse immunomodulatory effects, probiotics may decrease intestinal barrier permeability and cytokine-induced inflammation, consequently reducing gastrointestinal inflammation [85]. The colonisation of the intestine by probiotics may result in a bacterial population shift towards beneficial bacteria. These consist of bacterial-produced SCFAs and amino acids for supplementation, which have the potential to positively impact the gut microbiota population, microbial signals, and gastrointestinal symptoms. By influencing neuroendocrine and cytokine immune signalling, these modifications may affect the CNS and alleviate most of the symptoms associated with neurological ASD [86, 87].

### 2.6 Factor Influencing Microbiome Composition

Metagenomics, alternatively referred to as the catalogue of microorganisms and genes, is where the term ' microbiome' originated in 2019 when Joshua Lederberg invented it [88]. Following birth, the composition and diversity of the skin, nasal, and oral microbiomes resemble those of the vaginal and skin communities of the mother, with *Lactobacillus, Propionibacterium, Streptococcus,* and *Staphylococcus* being prevalent [89]. Humans are classified as holobionts due to the intricate interplay between host-derived cells and transient and stable microbial symbionts [90]. Ten times the number of nucleated cells, or 100 trillion microorganisms, comprise the average human body [91]. The gut microbiota composition is influenced to some extent by extracellular metabolites originating from the host and subsequently modified by microorganisms. Bile acids (BAs) comprise diverse metabolites modified by microbiota and originating from the host. These metabolites play a significant role in regulating the gastrointestinal microbiome [92]. Metagenomic sequencing (MGS) facilitates the differentiation between fungi, viruses, bacteria, and protozoa, thereby enabling the examination of predicted biological pathways and targeted analysis of bacterial genes [93]. Dysbiosis, an adverse modification in the composition and function of the intestinal microbiota, impacts the interaction between the host, microbiota, and the host immune system [94]. Human diseases, such as inflammatory bowel disease (IBD), irritable bowel syndrome, allergies, asthma, metabolic syndrome, and cardiovascular diseases, are increasingly linked to dysbiosis of the intestinal microbiota [95].

### 2.7 Physical Role of Gut Microbiome

The physiological benefits of intestinal microbiota to the host can be classified into three main domains: nutrition, immune development, and host defence. The gut microbiota plays a role in developing the immune system, nutrition, and host defense. It contributes to energy and nutrient provision, as well as the synthesis of vitamins K and B and short-chain fatty acids. Additionally, it facilitates immune system development by regulating T-cell responses, harmonizing TH1 and TH2 cells, and promoting IgA production. In addition, it aids in the host's defense by producing antimicrobial factors, including bacteriocins and lactic acid, and by resisting colonization [96].

Additionally, microbiota significantly influences digestion, nutrient assimilation, vitamin production, and metabolism. Indirectly, the gastrointestinal microbiome may influence an individual's response to cancer immunotherapy [93]. The gut microbiota comprises a complicated collection of microorganisms that inhabit the digestive tracts of various animals and humans, including insects. It contains the most species and the greatest variety of species among human anatomical structures [97]. Humans coevolve with the intestinal microbiota, and symbiotic interactions between the microbiota and the host are vital to human health maintenance [96].

### 2.8 Future Perspectives

Prominent population-based investigations, including but not limited to the Human Microbiome Project, MetaHIT, Lifelines DEEP, and the Flemish Gut Flora Project, have unveiled variables associated with gut microbiota composition [98]. These findings represent substantial advancements in the field of microbiome research. The impact of the microbiome on immune development commences with the initial colonisation of the gastrointestinal tract of the neonate [99]. The aberration of the typical human microbiota is correlated with an extensive spectrum of ailments, such as cancer, allergies, IBD, multiple sclerosis, obesity, autism, melancholy, and cardiovascular diseases [100]. The gut microbiota sustains a mutualistic association with the gastrointestinal mucosa in individuals who are in good health, thereby performing crucial metabolic, immune, and protective roles [101]. Probiotics, living bacteria, can provide health benefits when consumed [102]. In addition to affecting susceptibility to infectious diseases, chronic gastrointestinal disorders, such as Crohn's disease and irritable bowel syndrome, are also influenced by the microbiome [103].

## 3 THE EMERGING LINK BETWEEN GUT MICROBIOME AND AUTISM

GBA is a bidirectional neural network that links the enteric and central nervous systems. It establishes a connection between the affective and cognitive centres of the brain and the peripheral intestine *via* neuroimmunoendocrine mediators [104].

### 3.1 Overview of Studies Suggesting Microbiome Difference in ASD Individuals

Psychobiotics, such as *Bifidobacterium* and *Lactobacillus bulgaricus*, have the potential to decrease glutamate excitotoxicity, a critical component in rodent ASD models induced by oral propionic acid ingestion. By upregulating the GABA signalling pathway, decreasing oxidative stress, and restoring the damaged GABA signalling system, they may be capable of producing therapeutic effects [105]. The intestinal microbiota comprises a diverse array of bacterial species that establish mutualistic associations with the host and, under typical conditions, preserve a consistent composition; this stability gives rise to distinct human enterotypes [106]. Gabapentin, which functions as the principal inhibitory neurotransmitter of the CNS, is extensively distributed across the mammalian host and its receptors. Changes in GABAergic neurotransmission have been linked to a variety of central nervous system disorders, including pain, sleep disorders, and behavioural problems [107]. Quinones have also been recognised as growth promoters for bacteria in the human microbiome that were previously uncultured [108].

### 3.2 Exploration of Gut Microbiota's Influence on Neurodevelopment

The nervous system and gut microbiome are interdependent. From embryo to adulthood, the population and function of bacteria in the intestine impact nerve cell development by influencing synapse formation, axon and dendrite expansion, migration and projection of immature neurons, and the differentiation of neural stem cells [109]. The host's microbiota can aid in the expansion and function of microglia due to its complexity. Brain microglia, as innate immune cells, recognize histopathological alterations and transition into brain macrophages, thereby executing immune defence. Following injury of brain tissue, activated microglia have the ability to stimulate astrocytes. Proliferating inflammatory cytokines, including IL-6, TNF-alpha, IL-1β, and IFN-gamma, are secreted by activated microglia and astrocytes. These cytokines can potentially induce substantial disruptions in the immune system and impede the synaptic pruning mechanism [110].

#### 3.2.1 Mediators of Microbes-brain Interaction

Specific nutrients, which are subsequently assimilated by host cells with the aid of gut flora, exhibit noteworthy associations with neuronal activity. In addition to tryptophan and GABA, gut microbes generate monoamines, organic acids, and amino acids, such as serotonin, histamine, and dopamine. These compounds serve vital functions within the brain as neurotransmitters or precursors of neurotransmitters [111, 112].

#### 3.2.2 Role of Probiotics in the Gut-brain Axis

Probiotics are viable microorganisms that, when ingested in sufficient quantities, provide advantageous effects on the host's physiology. Although their complete effects remain unknown, they may regulate the host's immune system [113]. It has been reported that probiotic bacteria can modulate the immune response and promote a healthy gut environment.

#### 3.2.3 Role of gut Microbiota

A sufficient quantity of probiotics can improve health when ingested. However, further investigation is required to determine their potential to regulate the host's immune system, among other effects on host physiology [114, 115]. The gastrointestinal microbiome may influence neurotransmitter renewal, production, and expression, including acetylcholine, brain-derived neurotrophic factor, serotonin, dopamine, norepinephrine, and epinephrine [116]. Supporting the integrity of tight junctions and the intestinal barrier impacts the functions of the HPA axis and ANS. Specific probiotics modulate the activity of intestinal sensory afferents and the ENS.

Further, metabolites derived from bacteria in the gut have the potential to impact the brain. For instance, enterochromaffin cells in the intestine generate 90% of the body's 5-HT [117]. Modifying the microbiome's functionality is possible, either internally to a host or *via* transmission to its offspring (Fig. **3**) [118, 119].

### 3.3 Factors Influencing Gut Microbes

#### 3.3.1 Environment

The latency early life regulation (LEARn) model, recently introduced as an epigenetic framework for disease development, posits that early exposure to environmental stressors, including metals, nutritional imbalances, and variations in maternal care, among others, can alter the lifelong expression of genes associated with specific disorders [118, 119].

#### 3.3.2 Diet, Microbes, and Health

The intestinal microbiota's availability and utilisation of energy sources influence their diversity and abundance. An illustration of this is how a shift from a low-fat, high-sugar diet that is abundant in plant polysaccharides to a high-fat, low-sugar diet can impact the structure of microbiome communities, alter the representation of metabolic pathways, and modulate the expression of microbiome genes [120]. Enzymes required to degrade most complex carbohydrates and plant polysaccharides are absent in humans. As a result, following their passage through the upper gastrointestinal tract, microorganisms metabolize these polysaccharides in the colon. They generate water, gases, including carbon dioxide and methane, and metabolites, including volatile fatty acids [121].

#### 3.3.3 Mode of Delivery

A wide range of microorganisms, which participate in colonizing the gut microbiome, come into contact with infants during childbirth. The mode of delivery has a substantial impact on the intestinal microbiota of the infant. Surprisingly, the gut microbiota of the neonate closely resembles that of the infant at birth. Vaginal birth results in the inoculation of the neonate with vaginal microbiota, which is distinct from the skin microbiota that is encountered during cesarean section [122].

#### 3.3.4 Infant Feeding Method

Breast milk is the best food for infants since it meets all of their nutritional and physiological needs. In addition to protein, fat, and carbohydrates, it comprises immunoglobulins, endocannabinoids, and lipids [123]. Breast milk is not sterile, as evidenced by the presence of 600 distinct species of bacteria, some of which are beneficial (*Bifidobacterium breve, Bifidobacterium adolescentis, Bifidobacterium longum, Bifidobacterium bifidum*, and *Bifidobacterium dentium*) [124].

#### 3.3.5 Antibiotics

Antibiotic treatments have the capacity to affect the host-associated microbial communities in the intestine in addition to pathogenic microorganisms. Due to their comprehensive spectrum of action, most antibiotics are effective against a wide range of diseases. On the contrary, antibiotics have an adverse effect on pathogenic organisms and closely related microbiota members, which persists even after antibiotic use has ceased. This has a detrimental influence on the gut microbial community [125].

#### 3.3.6 Genetics

The quantity of particular bacteria in the gastrointestinal microbiota is also influenced by the host's genetic makeup, which, in turn, impacts the metabolism and health of the host [126]. Nevertheless, there is a lack of genome-wide investigations that have identified precise genes and pathways that regulate the intestinal microbiome's composition [126]. Certain immune system alleles are associated with inflammatory bowel disease [127-129].

### 3.4 Relationship between ASD and Gut Microbiota

Gastrointestinal issues are commonly reported in individuals with ASD. Research has found that there is a greater prevalence of gastrointestinal disorders, including constipation (20%) and diarrhoea (19%), among children with ASD in comparison to their typically developing sibling group (23%). Moreover, in ASD patients, severe behavioural manifestations, such as aggression, self-harm, and anxiety, may co-occur with prevalent gastrointestinal symptoms [130]. Additionally, variations in microbiota levels, such as *Sutterella, Prevotella*, and *Bifidobacterium*, have been observed in individuals diagnosed with ASD [131]. Further, evidence links ruminococcus torques to functional gastrointestinal disorders in ASD individuals [132].

### 3.5 Brain IL-6 Elevation and Autism

High levels of IL-6 are detrimental to long-term synaptic plasticity, influencing learning and memory in both physiological and pathological ways, according to many studies [133]. The number of primary dendrites, nodes, overall dendritic length, and neuron survival are all drastically diminished by IL-6. In cultured cerebellar granule cells, increased IL-6 levels alter the adhesion and migration of neural cells and disrupt the balance between excitatory and inhibitory circuits [134]. This is significant due to the clinical and molecular associations that exist between autism and schizophrenia [135]. An increase in IL-6 stimulates the formation of mushroom-shaped dendritic spines [136]. It significantly prolongs the length of these spines, providing additional evidence that IL-6 elevation may facilitate the initiation of neuronal excitation [137]. Elevated IL-6 levels impair synaptic plasticity, impacting learning and memory. They reduce dendrite numbers, nodes, length, and neuron survival. IL-6 disrupts neural cell adhesion and migration, upsetting the excitatory-inhibitory balance. Clinical links exist between IL-6, autism, and schizophrenia. Increased IL-6 promotes the formation of mushroom-shaped dendritic spines, extending their length and possibly facilitating neuronal excitation.

### 3.6 Exploration of the Bidirectional Communication between Gut and Brain

The “brain-gut axis” establishes a bidirectional link between the colon and the CNS using neurons, hormones, and immunological signals [138, 139]. Microbes in the gut function as both targets and sources of signals along the gut-brain axis [140, 141]. Emerging evidence suggests a connection between inflammation, a gene, and microbes. It has been observed that susceptible, infected individuals produce more TNF-alpha and other cytokines [142]. TNF-alpha induces melancholy in humans and anxiety in animals. TNF-alpha further stimulates inflammation as a cytokine with systemic and local effects [143].

### 3.7 Microbiota Gut-brain axis in Neurotransmitter

The gut microbiota of individuals in good health is composed primarily of bacteria belonging to the phyla *Bacteroides* and *Firmicutes* [144]. It influences the host through metabolic, neuroendocrine, immunological, and neurological pathways [25, 145]. By modulating serotoninergic, noradrenergic, dopaminergic, glutamatergic, and GABA-ergic neurotransmission, the gastrointestinal microbiota can affect brain function [19, 146]. Microbiota can either generate these neuroactive compounds or influence neurotransmitter synthesis and metabolism. Serotonin-producing bacteria, for instance, consist of *Streptococcus, Candida, Escherichia*, and *Enterococcus* [147].

## 4 THE MECHANISM OF THE INTERACTION BETWEEN THE MICROBIOTA AND BRAIN

Gnotobiotic and germ-free animal models are essential for studying gut microbiota regulation and the gut-brain axis. However, they are inappropriate for human usage due to physiological and metabolic differences. Piglets are the preferred model for studying host-microbe interactions [148, 149]. Notwithstanding the magnitude of research, the precise aetiology of ASD remains unknown. Existing knowledge indicates that environmental and genetic risk factors interact synergistically to contribute to ASD. Previously recognised for its ability to regulate gene-environment interactions, the human microbiome is now thought to play a role in the development of ASD *via* alterations in gut microbiota and its metabolites, which may influence associated immunological and gastrointestinal complications [150].

### 4.1 Microbiome-associated Maternal Risk Factors: ASD-associated Gut Microbiome Alteration

Maternal microbiome dysbiosis, which can be inherited from birth, may arise from environmental hazards or hereditary influences. Epidemiological studies conducted on humans and animals have presented evidence indicating that a high-fat diet and maternal exposure to metabolic disorders can elevate the risk of neurodevelopmental and behavioural disorders in offspring [151-153]. The decreased levels of genera, such as *Prevotella, Corprococcus*, and *Veilonellaceae*, which are responsible for carbohydrate ingestion and fermentation, are in the intestinal microbial compositions of children with ASD. Analyses of faecal microflora in children diagnosed with regressive autism indicated elevated concentrations of *Firmicutes* and reduced levels of *Bacteroidetes, Actinobacteria*, and *Proteobacteria* [154].

### 4.2 Immune System Modulation and its Effect on Neurodevelopment

Neuro-intestinal communication is possible *via* immunological pathways. Research has identified elevated concentrations of pro-inflammatory cytokines, such as IL-1β, IL-6, IL-8, and IL-12, in individuals diagnosed with ASD [155]. ASD children exhibit peripheral immune abnormalities, such as altered cytokine profiles of T helper cells, reduced lymphocyte count, modified T cell reactions, unbalanced levels of serum immunoglobulins, activated natural killer cells, increased monocyte responses, and elevated complement components [156-162]. Cytokines have the potential to affect neurodevelopment in diverse ways. For instance, the release of IL-1 during perinatal hypoxia has been linked to adverse neurological outcomes [163]. Meanwhile, TNF-alpha exerts a developmentally specific influence on dopaminergic differentiation or neuron apoptosis [164].

### 4.3 Microbial Metabolites and their Influence on Neurotransmitter Pathway

An increasing number of studies are linking alterations in gastrointestinal microbiota to various pathogenic pathways and neuropsychiatric disorders. Pathogenic pathways in severe neurodegenerative diseases have been linked to alterations in the gut microbiota; neuroinflammation induced by metabolites of dysbiotic microbiota is a significant contributing factor [165-168]. Persistent neuroinflammation is facilitated by the secretion of proinflammatory cytokines and substances, such as TNF-, IL-6, and MCP-1, by activated microglia [169, 170]. Chronic inflammation and a reduction in the integrity of intestinal epithelial cells are the primary effects of dysbiosis [171]. ASD is correlated with inflammation of the central nervous system; in children, elevated TNF- levels correspond to the severity of gastrointestinal symptoms (Table **1**) [172].

### 4.4 Role of Gut Microbiota in Inflammation and Oxidative Stress

Due to an imbalance between prooxidants and antioxidants, reactive nitrogen species (RNS) and reactive oxygen species (ROS) can induce cellular injury. This state is referred to as oxidative stress [173]. During electron transfer chain processes within mitochondria, ROS are generated as intermediates and byproducts of the energy production cycle [174, 175]. The preservation of oxidative equilibrium is vital [176]. A disruption in this equilibrium may occur when the capacity of the mitochondria is impaired due to persistent oxidative stress [177]. Under specific conditions, oxidized cofactors (NAD^+^ and FAD) are reduced to their reduced forms (NADH and FADH2) *via* β-oxidation, providing electrons to the respiratory chain. Maintaining an equilibrium between electron inflow and efflux within the mitochondrial respiratory chain (ETC) inhibits the buildup of reactive oxygen species (ROS) [178]. Among other alterations, oxidative stress can potentially induce lipid peroxidation, DNA and protein oxidation, inflammation, modifications in the immune response, decreased DNA methylation, and epigenetic dysregulation [179]. Although NO appears to have neuroprotective effects at low to moderate concentrations, its excessive production can oxidatively damage neurons and impair their functionality [180]. Propionic acid (PPA), which is secreted by the gastrointestinal microbiome and has both harmful and beneficial effects on mitochondria, might play an important role in individuals with ASD.

PPA has additionally been associated with the development of ASD [181]. The principal origin of ROS during mitochondrial activity is oxidative processes, which are intricately linked to, among other things, endoplasmic reticulum stress (ER stress), peroxisome, and proteasome activity [182]. ROS production induced by ER stress is critical for mitochondrial dysfunction [183]. Usually, the capacity of the cell to produce ROS/RNS and its ability to act as an antioxidant are in a state of dynamic equilibrium [184]. Superoxide is a major precursor of harmful free radicals, lipoperoxides, and hydroperoxides and is the main result of molecular oxygen reduction [185]. Emerging studies indicate a potential association between endoplasmic reticulum (ER) stress and the function of mitochondria, as suggested by the regulation of MAM proteins by the guanosine triphosphatase (GTPase) Rab32. This association may result in neurological complications and mitochondrial dysfunction [186].

## 5 INVESTIGATING THE GUT-BRAIN CONNECTION

Several studies have investigated how nutrients modulate gut-brain interactions and impact the luminal environment. A study also compared the effects of intragastric L-tryptophan (L-Trp) and L-leucine (L-Leu) on brain networks and the secretion of glucose and insulin [187].

### 5.1 Metabolic Pathway

The metabolic pathway, which involves the host's microbiome, epigenome, and metabolome in the production of neurotransmitters and metabolites, maybe the mechanism by which dysbiosis of the intestine is associated with ADHD. It has been demonstrated that the microbiota produce neurotransmitters associated with ADHD symptoms. *Bifidobacterium* species, serotonin produced by enterochromaffin cells in the intestine, and serotonin produced by species of *Lactobacillus, Streptococcus, Escherichia, Morganella, Klebsiella*, and *Hafnia* are all involved in the regulation of gut-brain communication. Furthermore, it has been observed that *Escherichia, Bacillus,* and *Bifidobacterium* species can produce GABA [188-190].

### 5.2 Vagus Nerve Pathway

As the principal nerve of the parasympathetic nervous system and the tenth cranial nerve, the vagus nerve regulates the immune system, digestion, heart rate, and mood. It connects the CNS with the gastrointestinal tract through separate and distinct fibers. Mood and anxiety disorders are treated using monoamines, whose activation in the brainstem's monoaminergic systems is influenced by vagal fibers in the stomach [191].

### 5.3 Immune Pathway

Research indicates that atopic diseases, such as eczema, allergic rhinitis, atopic dermatitis, and autoimmune diseases, are more prevalent in individuals with ADHD. Children with these atopic disorders have a 30–50% higher likelihood of developing ADHD later in life compared to those who are not atopic [192, 193]. Furthermore, several studies have found a link between ADHD symptoms and elevated levels of proinflammatory cytokines in the blood, including interleukin-13 (IL-13), IL-6, and TNF-α [194, 195]. Numerous investigations support the theory of immune involvement in ASD by demonstrating anomalies in the peripheral immune system. Additionally, the brains and cerebrospinal fluid (CSF) of ASD patients show immune abnormalities, such as neuroinflammation and the activation of microglial cells and the innate neuroimmune system [196].

### 5.4 Diagnostic Criteria and Neuroimaging Advances in ASD

The DSM's diagnostic standards for ASD, upheld by the American Psychiatric Association, state that the two main criteria for diagnosing ASD are deficits in social communication/interaction and the presence of restricted and repetitive patterns of behavior (DSM-5, 2013 edition) [197]. Grey matter volume, used as a structural factor for classification in a study of autistic children and adolescents, showed high discriminatory power between clinical and control groups in typical network regions (posterior cingulate cortex and medial temporal lobes: 92%, medial prefrontal cortex: 88%) using SVM searchlight classification [198]. The posterior cingulate's morphometric abnormalities, identified in an earlier study on adults with ASD, suggest that this cortical midline region may be a site of dysfunction across the lifespan. A recent survey of children employed a novel technique called multi-kernel SVM, combining regional (cortical thickness, grey matter volume) and interregional (morphological change patterns between pairs of ROIs) features to achieve 96% classification accuracy (Table **1**) [199-214].

### 5.5 Microbial Signatures as Potential Diagnostic Markers for ASD

Our study demonstrated that examining the gut and oral microbiomes can differentiate ASD patients from controls. Analysis of dysbiosis markers and differentially expressed taxa revealed three potential biomarkers: the gut *Firmicutes/Bacteroidetes* ratio, *Parvimonas* in saliva, and *Butyricimonas* in the gut [215]. This aligns with the study that found lower levels of *Butyricimonas* in the gut of ASD patients compared to controls [216]. These findings could potentially influence the social and cognitive deficiencies in autism. The ultimate goal is to identify biomarkers that are easy to use, affordable, non-invasive, and accessible in body fluids like blood or urine. This is because multi-omics approaches have the potential to clarify the pathobiology of ASD and identify novel biomarkers for improved diagnosis and therapeutic monitoring.

### 5.6 Autism Spectrum Disorder

(A) Molecular Traits: Abnormal growth of neurons, changes in neurotransmitters, dysfunction of the mitochondria, oxidative stress, neuroinflammation, and dysregulation of the immune system are significant molecular traits observed.

(B) Biomarkers: Genetic polymorphism, transcription factors and mRNA, gut-host and microbiota proteins, interleukins, and metabolites in blood, urine, and faeces have been identified as biomarkers [217].

(C) Environmental Factors: Environmental factors may influence the developing fetus's brain activity directly or indirectly. This can be through the activation of neuroinflammatory responses and gene dysregulation, thought to be caused by the activation of the mother's immune system and communication through the placenta [218, 219].

(D) Epigenetic Factors: In recent decades, epigenetics, which involves the impact of environmental influences on genes, has become a primary focus in studying the aetiology of ASD. Earlier research methods, which considered genes and environmental factors separately, lacked an integrated approach. 'Epigenetic' refers to non-genetic factors that alter a gene's phenotypic expression without changing the original DNA sequence. These modifications are heritable [220].

Biomarkers must be quantifiable, specific to the disorder, and reliable within and across individuals. Saliva, urine, and blood are biological materials used for biomarker assessment. Neuromarkers, based on CSF and MRI scans, are gaining interest for their potential applications in identifying risk factors, early diagnosis, predicting disorder severity, and tailoring treatments. Despite numerous proposed biomarkers for autism, none have been validated for clinical use [221].

### 5.7 Identification of Specific Microbiota Alteration in ASD Individual

The criteria outlined in the DSM-5 are exclusively predicated on clinical symptoms and do not incorporate objective laboratory testing. Currently, the diagnosis of ASD is determined by these criteria. However, combining oral and gastrointestinal microbiome markers can help diagnose and screen individuals with ASD, identify clinical or subclinical subgroups, and predict therapeutic responses. This method could enhance personalized treatment and outcomes, particularly when considering medications or probiotic therapy in conjunction with anti-inflammatory and probiotic treatments [216].

Significant alterations have been observed in the composition of the gastrointestinal microbiota between individuals with ASD and healthy controls. Pyrosequencing of faecal DNA samples revealed distinct and less diverse gut microbial compositions in individuals with ASD. The observed taxa, including *Prevotella, Corprococcus*, and *Veilonellaceae,* are essential for fermentation and carbohydrate assimilation [153]. *Firmicutes* exhibited a relatively higher abundance in children diagnosed with regressive autism compared to *Bacteroidetes, Actinobacteria*, and *Proteobacteria* at the phylum level. In addition, the *Firmicutes/Bacteroidetes* ratio was elevated, and the abundance of *Lactobacillus* and *Desulfovibrio* species was more remarkable in individuals with ASD [222, 223]. Research on ASD-related gastrointestinal microbiome changes is limited, with many bacterial species affected and their physiological functions unknown. Functional changes may be influenced by xenobiotics, microbial infections, and dietary patterns, which affect the typical microflora profile (Table **2**) [224].

### 5.8 Correlation between Behavioural Symptoms and Gut Microbiota

Cortisol, regulated by the HPA axis, has significant effects on the gut both locally and systemically. It also influences immune cell function, gastrointestinal microbiota composition, and intestinal barrier function. Changes in the microbiota and probiotic supplements can alter circulating cytokine levels, impacting cerebral function. The gastrointestinal microbiota impacts brain function through vagus nerve communication, systemic tryptophan levels, and the production of neuroactive bacterial metabolites from dietary fibre, such as SCFAs [225]. Two studies suggest a correlation between ASD gastrointestinal symptoms and gut microbiota, with prebiotic supplementation significantly affecting these microbiotas [226]. A positive correlation was observed between defecation frequency and Blautia wexlerae strain abundance (Table **2**) [227].

### 5.9 Microbiome-mediated Interventions in ASD: Clinical Evidence and Therapeutic Potential

Probiotic strains from *Bifidobacterium* (*e.g.,* B. *longum, B. breve,* and *B. infantis*) and *Lactobacillus* (*e.g.,* *L. helveticus* and *L. rhamnosus*) administered in doses ranging from 10^9^ to 10^10^ CFUs have been shown to ameliorate psychiatric disorder-related behaviors in human studies. Randomized controlled trials (RCTs) over two weeks demonstrated a reduction in pro-inflammatory cytokines (*e.g.,* TNF-α, IL-6, and IL-12) and an increase in anti-inflammatory cytokine levels (*e.g.,* IL-10). They enhanced natural killer (NK) cell activity, particularly in smokers [228]. Similarly, supplementation with *Lactobacillus salivarius* and *Bifidobacterium infantis 35624* (108 CFUs/day) in 362 subjects with ASD during an eight-week intervention (six weeks with placebo) improved scores related to bowel habit satisfaction and global self-assessment of irritable bowel syndrome (IBS) relief [229]. A combination of probiotics, including *Streptococcus thermophilus*, *Bifidobacterium breve, Bifidobacterium longum, Bifidobacterium infantis, Lactobacillus acidophilus,* and *Lactobacillus plantarum,* administered to 173 children with ASD as two packets/day in a five-month RCT, reduced systemic and intestinal inflammation while modulating circulating lipopolysaccharide levels and influencing brain-derived neurotrophic factor (BDNF) expression [230]. Another trial utilizing *L. casei, L. plantarum, L. acidophilus, and L. delbrueckii subsp. bulgaricus,* and three strains of *Bifidobacterium (B. longum, B. infantis*, and *B. breve*) in powder form (900 billion bacteria per packet) over 15 weeks in a placebo-controlled pilot study involving 13 children demonstrated improved mental health and reduced cortisol levels [227]. In another study, *Lactobacillus plantarum WCSF1* at a dose of 4.5×10^10^ CFUs per capsule/day, administered to 20–30 children with ASD in a double-blind, placebo-controlled crossover design study, significantly improved behavioral scores and stool consistency, increased *Enterococci* and *Lactobacilli*, and decreased *Clostridium* cluster XIVa [231]. A pilot trial involving *Lactobacillus plantarum PS128* (6×10^10^ CFUs) administered to 32 children with ASD over 28 weeks revealed significant improvements in Aberrant Behavior Checklist (ABC) and Social Responsiveness Scale (SRS) scores, along with marked clinical global impression (CGI) improvements [232]. Lastly, a probiotic blend containing *L. acidophilus, L. plantarum, L. paracasei, L. bulgaricus,* and *L. delbrueckii subsp*., administered at 8×10^10^ CFUs over four weeks with a four-month follow-up to 18–20 children, demonstrated a potential role in mitigating behavioral abnormalities associated with ASD [233]. A study found that microbiota transfer therapy, which consists of faecal microbiota transplant, colon cleaning, and antibiotic combination, significantly improved gastrointestinal symptoms and autism-related symptoms in 18 patients two years after therapy. To validate these results, further double-blind, placebo-controlled research is required [234]. The findings in studies highlight the promising therapeutic potential of probiotics in managing ASD and related psychiatric disorders by modulating gut microbiota, inflammation, and neurobehavioral outcomes.

## 6 POTENTIAL THERAPEUTIC APPROACH

A potential treatment strategy that targets the gut microbiome could be efficacious, considering the substantial evidence supporting the pivotal role of gut microbiota in the development of ASD. Diet has a considerable impact on the composition of the gastrointestinal microbiota, especially with regard to particular macronutrients. The memory-impairing and anxiety-inducing effects of Western-style dietary patterns may vary depending on the inflammatory state [235]. In contrast, supplementation with high doses of polyunsaturated fatty acids may alleviate depression in rodent models [236].

### 6.1 Probiotics and their Potential in Modulating the Gut Microbiome

The etymological origin of 'probiotic' is the Greek phrase 'pro bios,' which translates to 'for life.' There are ancient origins to the beneficial effects of lactic acid fermentation products on human health [237]. Élie Metchnikoff, a Nobel laureate and microbiologist, is credited with developing the original theory on the role and function of probiotic microbes [238]. Probiotics have been demonstrated to assist nutrient absorption and improve intestinal barrier function [23]. This is achieved by altering the intestinal immune system and the gastrointestinal microbiota composition. Probiotics administered orally have demonstrated efficacy in treating a range of gastrointestinal disorders, including infectious diarrhoea and IBS [239]. In addition to modulating the Th1 response and regulating immunological and inflammatory responses, probiotics stimulate the development of regulatory T cells. This modulation is achieved through two mechanisms: either by increasing the expression of anti-inflammatory mediators, such as IL-10 and β-TFGF, or by inhibiting the synthesis of pro-inflammatory cytokines like IL-12, TNF-α, and INF-α by dendritic cells [240, 241].

On the other hand, probiotics are more selective than prebiotics since prebiotics have minimal or no influence over which particular microbes undergo metabolism and proliferate when administered [242]. A symbiotic, which is a mixture of probiotics and prebiotics, might be more advantageous. Probiotics consist primarily of two bacterial genera, *Bifidobacterium* and *Lactobacillus*, capable of generating lactic acid. Probiotics may aid in treating various diseases (including AD, ASD, and epilepsy) and improve cognitive outcomes, according to some animal studies. Although probiotics have been tested on humans in limited clinical trials, their effectiveness in treating neuropsychiatric disorders remains uncertain [243]. It has been demonstrated that *Lactobacillus rhamnosus*, *Lactobacillus helveticus,* and *Lactobacillus fermentum* can ameliorate stress-induced memory impairment in rodents [244].

### 6.2 Dietary Intervention for Promoting a Healthy Gut-brain Axis

In recent times, scholars have initiated investigations into the potential impact of dietary interventions on individuals with ASD. In light of the fact that people with ASD frequently display challenging and selective eating behaviours, it is crucial to comprehend the possible physiological effects of dietary interventions [245, 246]. Further, it is imperative to consider dietary patterns that could potentially worsen gastrointestinal disorders and disrupt the gut microbiota equilibrium. The Mediterranean diet (MD), the ketogenic diet (KD), the specific carbohydrate diet (SCD), and the gluten-free/casein-free diet (GF/CFD) are the dietary strategies that have been the subject of the most scholarly investigation [247].

#### 6.2.1 Gluten-free/casein-free Diet (GF/CFD)

As one of the interventions for ASD, the GF/CFD diet mandates excluding all foods containing wheat, oats, barley, or rye. This includes pastries, flour, bread, rusks, pasta, and other confectionary items that contain these cereals. According to research involving healthy individuals, a gluten-free diet is associated with diminished beneficial gut flora populations, increased opportunistic pathogens, and immune-suppressing effects [248-250].

#### 6.2.2 Mediterranean Diet (MD)

The diversity of the intestinal microbiome is diminished in individuals who adhere to a Western diet, which is distinguished by its high content of refined grains, calories, sugar, and saturated fat. Additionally, this dietary pattern is correlated with a rise in the population of *Firmicutes* and *Proteobacteria* organisms and a decline in the quantity of *Bacteroidetes* members. Such alterations in microbial composition may affect the structure and function of neurons. The Mediterranean diet, unlike the unhealthy Western diet, is rich in fruits, vegetables, bread, nuts, seeds, olive oil, and PUFAs, moderately consuming dairy, eggs, white meat, and fish, and limiting potatoes, red meat, and sweets [251, 252].

#### 6.2.3 Ketogenic Diet

The ketogenic diet (KD) encourages the body to rely on fat as its primary fuel source due to a reduction in carbohydrate consumption during fasting. It functions as a dietary intervention in treating various neurological and psychiatric disorders [253]. Scholarly investigations have centered on the efficacy of KD in mitigating symptoms associated with epilepsy, ASD, obesity, and AD. Nonetheless, research regarding the impact of KD on gastrointestinal microbiota is scarce. While clinical trials have demonstrated alterations in the gut microbiome subsequent to KD, the results of these studies diverge. The observed variability could be attributed to the restricted sample sizes and brief KD implementation periods. Future studies should aim for larger sample sizes and longer KD periods to ensure safety and reliable results on intestinal microbiota influence, especially in infants and older individuals with neurological conditions. In addition, communication difficulties, heightened food sensitivity, and rigid behaviour, particularly in children with autism, may confound the implementation of KD [254].

#### 6.2.4 Specific Carbohydrates Diet

Initially devised by Sidney Haas during the 1920s, the Specific Carbohydrate Diet (SCD) was popularised by Gottschall for treating celiac disease [255]. SCD implements dietary restrictions on the consumption of complex carbohydrates, including disaccharides, such as lactose, maltose, sucrose, and starch, to inhibit the development of pathogenic intestinal microbiota and alleviate symptoms of malabsorption. The diet recommends consuming abundant monosaccharide-rich foods and vegetables, fruits, nuts, meats, eggs, and specific legumes (including those containing galactose, fructose, and glucose). The diet omits sugar, starches, such as cereals and potatoes, processed foods, and most dairy products [256]. In patients with IBD, SCD, a highly restrictive diet that temporarily restricts the consumption of the majority of disaccharides and carbohydrates, has been shown to alleviate intestinal symptoms [257].

### 6.3 Fecal Microbiota Transplantation (FMT) as a Novel Therapy Avenue

FMT is an innovative therapeutic approach that enables the transfer of faecal matter from one individual to another in order to transmit the microbiota of the donor. This provides an additional means of modifying the microbiome. Utilized in scientific investigations, this technique aims to ascertain whether an animal can acquire a disease phenotype from the microbiota of a donor afflicted with a particular disorder, such as depression [258]. FMT therapy was investigated in one study involving eighteen individuals who were diagnosed with gastrointestinal issues, such as constipation, diarrhoea, indigestion, and abdominal pain, in addition to ASD [259]. The FMT significantly improved bowel movements in participants with chronic constipation and/or incontinence, resulting in a 26% decrease in abnormal diarrhoea and a 58% reduction in Gastrointestinal Symptom Rating Scale scores at a 2-year follow-up. Daily stool recordings and every subcategory of the GSRS showed progress. Furthermore, a positive correlation was found between percentage changes in scores on the Aberrant Behaviour Checklist (ABC), Social Responsiveness Scale (SRS), and Childhood Autism Rating Scale (CARS) and alterations in GSRS scores [260]. However, the correlation between daily stool records, an additional GI assessment, and behavioural severity did not reach statistical significance [261]. The delivery method differs among clinical trials, encompassing oral capsules, nasogastric, nasoduodenal, and colonoscopic routes. Treatment of severe infections caused by *Clostridium difficile* and inflammatory bowel diseases is advised with FMT. It alters the composition of the gastrointestinal microbiota considerably and potentially reverses the changes [262].

## 7 CHALLENGES AND FUTURE DIRECTIONS

### 7.1 Limitation of Current Research in the Field

An innovative strategy in the study of ADHD and ASD has emerged through the utilization of machine-assisted classification of neuroimaging data. This development holds considerable promise in the fields of diagnosis and treatment. Biomarkers can offer valuable insights into underlying behavioral symptoms and aetiology, improving behavior-based diagnoses in complex cases. They can also predict a child's development of a specific condition in utero or early childhood, identifying high-risk children before the onset of symptoms. Thus, biomarker testing is crucial for early detection and diagnosis [263]. While the primary outcome measure demonstrates a substantial effect size, conclusions about the potential impact and generalizability of the treatment are limited due to the small sample size and the broad spectrum of scores. Additional research into MDMA-assisted psychotherapy as a treatment for social anxiety disorder is required in light of these findings. Further, evaluating dosage response effects among subgroups was impossible due to the insufficient sample size of the study [264].

### 7.2 Exploration of Personalized Microbiome-based Therapy

#### 7.2.1 Additive Treatment

Microbial therapies, utilizing naturally occurring microorganisms, have been developed through genetic engineering to enhance host-microbe interactions and treatment efficacy [265]. The primary objective of microbiome research is to establish causal relationships and develop therapies that target the microbiome in order to predict the effects on host health and microbial communities. Most microbiome-based therapeutics focus on the prokaryotic components of the microbiome, manipulating the composition of gut microbes through exogenous delivery [266]. Alternatives to probiotics include prebiotics, compounds that alter the composition or function of the microbiome beneficially when ingested. Like probiotics, prebiotics represent a microbiome-based intervention strategy that lacks specificity, thus requiring additional research to elucidate their impact on various bacterial species comprehensively. FMT, an FDA-approved microbiome-based intervention, has succeeded significantly. In order to replace the disease-associated microbiome, FMT involves transferring a microbial community from a healthy donor to an afflicted recipient. FMT has demonstrated exceptional effectiveness in treating infections caused by *Clostridium difficile*, which occur frequently after antibiotic therapy [267].

## 8 ETHICAL CONSIDERATION IN MICROBIOME RESEARCH AND TREATMENT: AN OVERVIEW OF MORAL DILEMMAS

Early detection and intervention in ASD pose challenges to clinically sound therapies, including having clear reasons for action, knowing when to intervene, and accurately weighing benefits and drawbacks, requiring new models [268]. The ethical dilemmas at hand influence the way we perceive and treat individuals with mental illnesses and require families, patients, physicians, and society to make decisions. In addressing biological and psychosocial aspects, psychiatric medicine ought to integrate both by emphasizing the patient, who is the physical manifestation of the illness, as opposed to the disease itself [269].

### 8.1 Addressing Concerns Related to Experimental Treatment

In addition to its diagnostic and screening functions, the Childhood Autism Rating Scale (CARS) also includes a severity score. A total of fourteen items comprise this assessment, with one domain dedicated to the overall perception of autism. From 1 to 4, a higher score denotes more severe symptoms. The diagnostic threshold for autism is 30, while the range of total scores is from 15 to 60. Autism is classified as severe when values are elevated, whereas scores ranging from 30 to 36.5 indicate mild to moderate autism [270]. A wide range of interventions are available to tackle the multifaceted difficulties that are linked to ASD, which have an impact on both children and their families. These include behavioural or educational therapies, environmentally modified spaces, financial assistance, respite care, and alternative treatments. According to the results of a survey, parents applied seven out of 111 prescribed remedies to their children on average [271]. Parents usually believe that intervention for autism-related issues is of the highest priority due to the complexity of the situation. This sentiment is shared by the majority of parents whose children have severe health issues. Parents are regularly presented with abundant information that emphasizes the critical nature of early intervention to optimize their children's developmental trajectory and ensure more favourable long-term outcomes. This form of exposure significantly impacts the sense of urgency in autism cases [272].

## CONCLUSION

Research has established associations between gut microbiota and ASD symptoms, including gastrointestinal and socioemotional behaviour issues. These connections are mediated *via* immune, neuroendocrine, and metabolic pathways. Neuroimaging studies have also highlighted the interrelationships between microbiota and brain regions, including the amygdala and insula, implicated in various theories of ASD. Rodent studies lend credence to the notion that microbiota may play a role in ASD. However, further research is needed to understand the neural circuitry and mechanisms underlying this connection. Microbiome-based therapies aim to ascertain causal connections and devise therapeutic approaches that utilize the microbiome to generate foreseeable impacts on the host and microbial community. Current microbiome-based therapies focus on the prokaryotic component of the microbiome and use exogenous administration of living organisms to modify the gut's microbial composition. FMT is an FDA-approved microbiome-based intervention that has shown efficacy. Postbiotics are metabolic-based therapeutics designed to alleviate the detrimental consequences caused by overabundance, insufficiency, or dysregulation of metabolites in the signaling pathways downstream of the microbiome. Interdisciplinary cooperation is crucial in understanding and assisting people with autism. ASD has a global impact and requires collaboration among experts from various fields, including education, genetics, neuroscience, psychology, and technology. Collaborating with educators, therapists, physicians, and carers is critical for developing individualized, comprehensive strategies.

## Figures and Tables

**Fig. (1):**
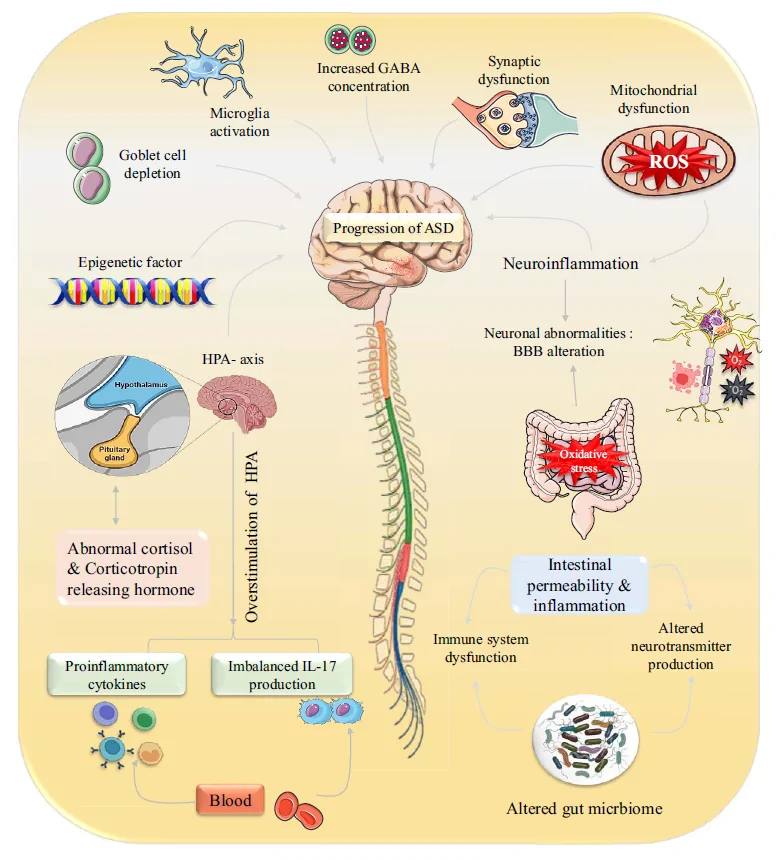
Involvement of gut-brain axis in the progression of autism spectrum disorder. The figure illustrates several interconnected factors involved in the progression of autism, including immune dysfunction, oxidative stress, environmental factors, goblet cell dysfunction, elevated GABA concentrations, synaptic dysfunction, mitochondrial dysfunction, alterations in the gut microbiome, and disrupted neurotransmitter production. These factors contribute to increased oxidative stress and intestinal permeability, often resulting in neuronal abnormalities, such as alterations to the blood-brain barrier (BBB). This disruption leads to neuroinflammation and highlights the involvement of the gut-brain axis in the progression of autism. Additionally, an imbalance in IL-17 production and pro-inflammatory cytokines in the blood can overstimulate the hypothalamic-pituitary-adrenal (HPA) axis, leading to the release of corticotropin-releasing hormone (CRH), further contributing to neuroinflammation and the gut-brain axis dysfunction in autism. **Abbreviations:** GABA: Gamma-aminobutyric acid, BBB: Blood-brain barrier, HPA: Hypothalamus pituitary axis, IL: Interleukin, 5-HT: 5 Hydroxytryptophan.

**Fig. (2):**
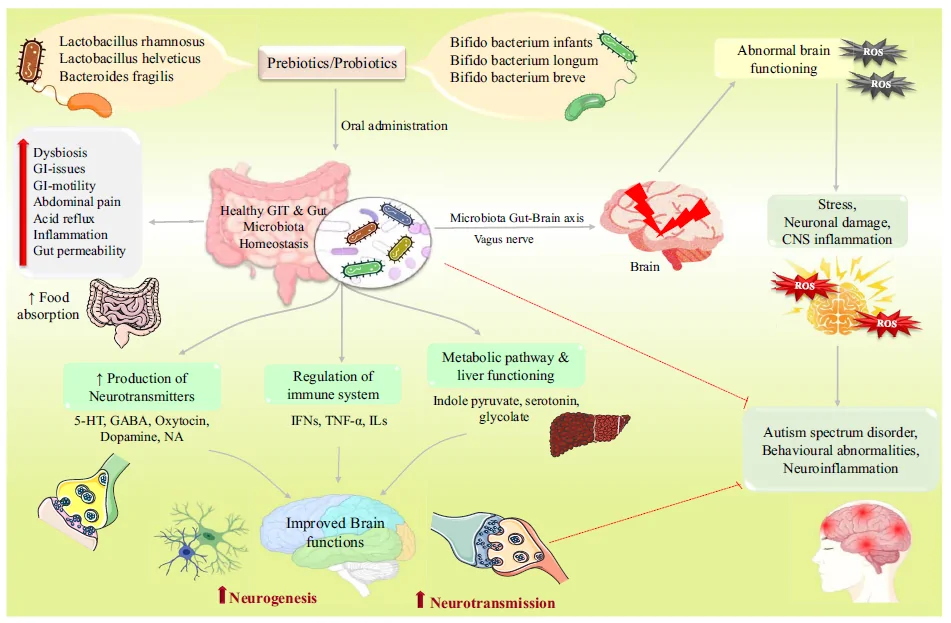
Probiotics used in the prevention of autism. Probiotic supplementation can improve gastrointestinal microbiota and the severity of Autism Spectrum Disorder (ASD) symptoms in children aged five to nine. Probiotics can act as anti-inflammatory agents and alter neuroactive compounds, potentially disrupting the balance between excitatory and inhibitory neurotransmission in autism. Psychobiotics stimulate neurotransmitters, affecting enteric nervous system communication and barrier functionality. Probiotic colonization may shift bacterial populations towards beneficial bacteria, potentially ameliorating symptoms associated with neurological ASD. **Abbreviations:** IL: Interleukin; NA: Noradrenaline; GIT: Gastrointestinal tract; GIM: Gastrointestinal motility; GP: Gut permeability; ROS: Reactive oxygen species. ↑: Increase, ↓: Decrease.

**Fig. (3):**
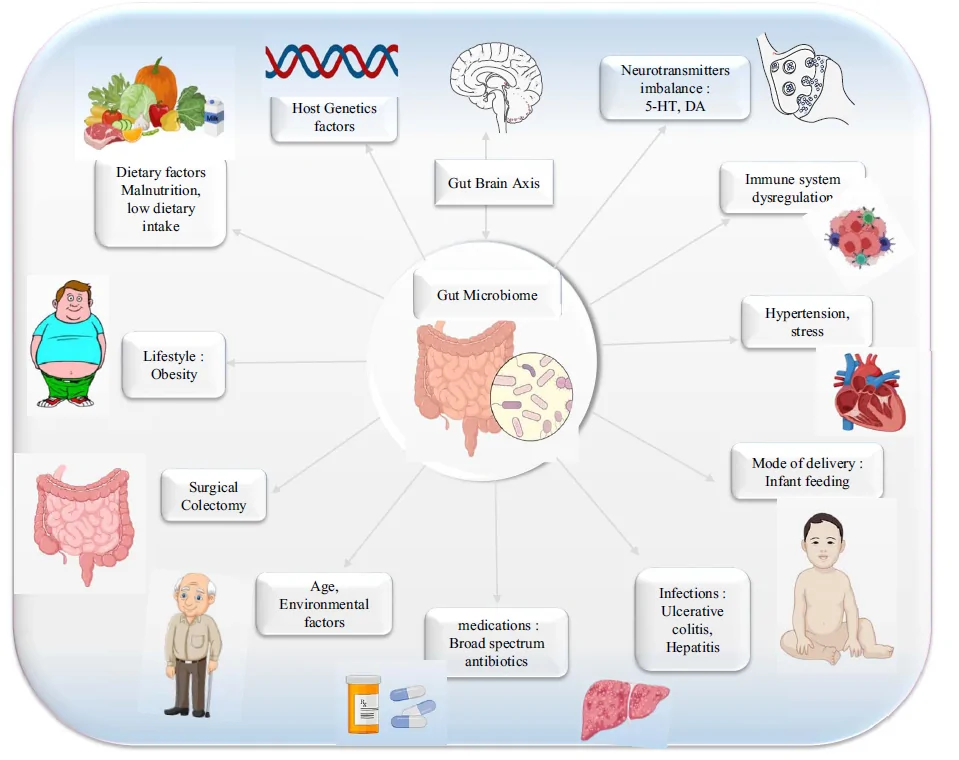
Factor influencing gut microbes. This figure depicts the neurotransmitters, such as serotonin, dopamine, norepinephrine, epinephrine, BDNF, and acetylcholine, along with other factors, such as genetic factor and environmental factors, age-related factors, lifestyle, obesity, dietary factor, low dietary intake, surgical colectomy, drug, and medication, such as cyclophosphamide, broad-spectrum antibiotics, such as dextran sulphate, immune system dysregulation, mode of delivery, infant feeding method, infection ulcerative colities, inflammatory bowel diseases, hepatitis, hypertension, and stress. **Abbreviation:** DA: Dopamine: 5-HT: serotonin; IBD: Inflammatory bowel disease; BDNF: Brain-derived neurotrophic factor.

**Table 1:** Therapeutic effects of probiotics in neurological diseases.

**S. No**	**Disease Condition**	**Animal Model**	**Species Used (Species WT)**	**Probiotics**	**Probiotics (Dose and Route)**	**Experimental Finding**	**References**
1	Acute pancreatitis	Rat model	Sprague-Dawley rats; 250 to 350 grams	*Lactobacillus casei* (W56), *Lactobacillus salivarius* (W24), and *Lactococcus lactis* (W58)	The volume of 1.0 ml contained 5 × 10^9^ colony-forming units (CFU) bacteria.	↓Pathogens in the duodenum and ↓ acute pancreatitis-induced pathogen overgrowth in the small bowel.	[200]
2	Alzheimer's Disease	Animal model of Alzheimer's disease	Male Wistar rats weighing 200–250 g	*Lactobacillus acidophilus, Bifidobacterium bifidum*, and *Bifidobacterium longum 1714*	The capsule consisted of 500 mg of the bacteria mixture with a total CFU of 15 × 10^9^ dissolved in drinking water and administered *via* intragastric gavage.	It + impaired cognition. It suppresses LTP in animals with Alzheimer's disease. It substantially restored synaptic plasticity.	[201]
3	Colorectal cancer	Colorectal cancer murine model	BALB/c mice weighing 22–24 g	*Lactobacillus casei* variety *rhamnosus* (Lcr35)	Diluted in sterile saline and administered by oral gavage. 1 × 10^3^ CFU of the probiotics. The effects of Lcr35 (1 × 10^7^ CFU/daily).	It↓ IL-1β, IL-6, TNF-α, and IL-10 mRNA expression in the jejunum of mice.	[202]
4	Obsessive-compulsive disorder	OCD model	BALB/cJ house mice	*L. rhamnosus*	Physiological saline by oral gavage (1/day).	A probiotic can ↓ OCD-like behaviors in an animal model.	[203]
5	Parkinson’s Disease	Rat model of PD	Naive adult male Sprague Dawley rats	*Lacticaseibacillus rhamnosus HA-114*	Distilled water at a concentration of 10^9^ colony forming units per milliliter (CFU) 10^8^ CFU.	Improvement in cognitive function with the potential to ↓ oxidative stress, metabolic state, and inflammatory processes.	[204]
6	Parkinson’s Disease	PD animal model	MitoPark PD mouse	*Bifidobacterium bifidum*, *Bifidobacterium longum, Lactobacillus rhamnosis, Lactobacillus rhamnosus GG, Lactobacillus plantarum LP28,* and *Lactococcus lactis subsp. Lactis*	10^10^ (CFU/mouse/day) dissolved in sterilized distilled water	MitoPark PD model, which features neuronal dopamine loss caused by mitochondrial dysfunction as its characteristic pathology.	[205]
7	Depression	Animal models of depressive-like behavior	Adult male Swiss mice (25–35 g)	*Komagataella pastoris KM71H*	8 log UFC∙g-1 /animal, intragastric route	Antidepressant-like effect of the administration of *K. pastoris KM71H* could be attributed, in part, to its antioxidant capacity.	[206]
8	Alzheimer’s Disease (AD)	Animal models used in peripherally induced neuroinflammation and neurodegeneration	Male Wistar rats (200–250 g)	*L. helveticus R0052 and B. longum R0175*	10^9^ CFU/ml/rat given orally	Probiotic pretreatment prevented the stress-induced ↓ in neurogenesis and abnormal synaptic plasticity and restored tight junction barrier integrity.	[207]
9	Pancreatic cancer	Mouse model	Transgenic mice	*Lactobacillus paracasei GMNL-133* and *Lactobacillus reuteri GMNL-89*	GMNL-133: GMNL-89 in a 1:1/10 ratio (1.64 × 10^7^ CFU/0.02 kg (0.2 ml) by oral gavage)	Intestinal microbiota, immunomodulation, ↓inflammation, inhibitory effects on cancer, ↑nutrient absorption, production of short-chain fatty acids, ↑ pathogen replication.	[208]
10	Intestinal injury	Mouse model	Male 6-8 weeks-old C57BL/6J mice	*Lactobacillus acidophilus LA85, Lactobacillus rhamnosus LRa05, Lactobacillus plantarum Lp90, Bifidobacterium longum BL21* and *Bifidobacterium lactis BLa80*	0.1 g of powder, containing 10 billion colony-forming units dissolved in phosphate-buffered saline and was orally gavage-administered	↑ Shortening of villus height, ↑ no. of goblet cells, ↑no. of surviving crypts across the intestinal section, and Ki67 positive cells in each crypt. ↑proliferation of intestinal crypt cells in radiated mice.	[209]
11	Autoimmune thyroiditis	Models of autoimmune thyroiditis	Female mice (4–6 weeks old)	*Lactobacillus rhamnosus HN001(HN001)* and *Bifidobacterium lactis HN019 (HN019)*	1– 1.5× 10^8^ cfu/mouse/day for 5 to 9 weeks	↑ immune function, ↑innate and adaptive immune responses.	[210]
12	Multiple Sclerosis	Animal models of MS	Mouse	*L. plantarum A7, L. delbrueckii, DSM 20081, L. paracasei DSM 13434, L. plantarum DSM 15312,* and *L. plantarum DSM 15313*	10^9^ CFU/mL, used dosage was 0.6–5 × 10^8^ –10^9^ colony-forming units (CFU) for 22 to 50 day, oral gavage	↓ progression of the disease and ↑clinical and histological signs of EAE in mesenteric lymph nodes (MLNs).	[211]
13	Depression	Naïve adult mice	Mice	*L. plantarum PS128*	10^9^ CFU/mouse/day by gavage	Locomotor activity Anxiety-like behaviors in naive mice Y. Depression-like behaviors in ELS mice Y. ↓ Corticosterone levels in ELS mice Y TNF-α and IL-6γ	[212]
14	Inflammatory bowel diseases	Murine models of IBD	BALB/c mice	*Lactobacillus rhamnosus GG*, *L. rhamnosus LC705*, *Bifidobacterium breve 99*, and *Propionibacterium freudenreichii ssp.*	Intragastric administration of 10^8^ or 10^9^ colony-forming units (cfu)/mL	↓ IL-12 and IFN-γ production	[213]
15	Autism	ASD animal model	Mice	*B. fragilis NCTC 9343*	10^10^ CFU of freshly grown B. fragilis, or vehicle, in 1.5% sodium bicarbonate was administered in sugar-free applesauce over standard food pellets	↑ Gut barrier integrity, normalized gut microbiota, and reversed ASD-related behaviours.	[214]

**Note:** This table represents several studies indicating the therapeutic effects of probiotics in various neurological diseases (↑: Increase,↓: Decrease,+: Stimulate, –: Inhibited). **Abbreviations:** CFU: Colony forming unit, MS: Multiple sclerosis, PD: Parkinson's disease, IBDs: Inflammatory bowel diseases, MLNs: Mesenteric lymph nodes, and ASD: Autism spectrum disorder.

**Table 2:** List of Probiotics Supporting Gut Health in Individuals with ASD.

**S.no**	**Probiotics Involved**	**Dose and Route**	**Types of Patients**	**No of Patients**	**Experimental Schedule and Types**	**Experimental Finding**	**References**
1	*Bifidobacterium* (*e.g.,B. longum*, *B. breve*, and *B. infantis*) and *Lactobacillus* (*e.g.,* *L. helveticus* and *L. rhamnosus*),	Doses between 10^9^ and 10^10^ colony-forming units	Psychiatric disorder-related behaviors	15 humans	2 weeks; only randomized controlled trials were included.	↓Pro-inflammatory cytokines (TNF-α, IL-6, and IL-12), ↑cytokines (*e.g.,* IL-10), and ↑natural killer cell activity in smokers	[228]
2	*Lactobacillus salivarius* and *Bifidobacterium infantis* 35624	(10^8^ cfu/day)	Autism Spectrum Disorders	362 subjects	8-week placebo for a six-week intervention period	↑included scores for (1) bowel habit satisfaction and (2) global self-assessment of IBS relief	[229]
3	*Streptococcus thermophilus, Bifidobacterium breve, Bifidobacterium longum, Bifidobacterium infantis, Lactobacillus acidophilus,* and *Lactobacillus plantarum*	2 packets/ day	ASD	173 children	5 months randomized controlled trial	↓Reduction in systemic or intestinal inflammation. ↑The levels of circulating lipopolysaccharide and modulate the levels of brain-derived neurotrophic factor	[230]
4	*L. casei*, *Lactobacillus plantarum, Lactobacillus acidophilus,* and *Lactobacillus delbrueckii subsp. bulgaricus*, three strains of bifidobacteria (*B. longum, Bifidobacterium infantis,* and *Bifidobacterium breve*)	In powder packets, each containing 900 billion bacteria;	ASD	Thirteen children	15-week placebo-controlled pilot trial	Probiotic intake is linked to better mental health. ↓Cortisol levels	[227]
5	*L. plantarum* WCSF1	4.5×10^10^ CFU per capsule per day	ASD	20–30 Children	A double-blind, placebo-controlled, crossover designed study	Improve behavioral scores and stool consistency, ↑*Enterococci* and *Lactobacilli* group, ↓*Clostridium* cluster XIVa	[231]
6	*Lactobacillus plantarum* PS128	(6 × 10^10^ CFUs)	ASD	32 children	28-weeks randomized, double-blinded, placebo-controlled pilot trial	Itꜛ↑ ABC and SRS scores and significant ↑in CGI improvement among those receiving probiotics	[232]
7	*Lactobacillus acidophilus, L. plantarum, L. paracasei, L. bulgaricus, and L. delbrueckii subsp.*	8×10^10^ CFUs	ASD	18-20 children	A 4-week study, followed by a four month follow-up	A possible role of probiotics in the improvement of behavioral abnormalities associated with ASD	[233]
8	*Bifidobacteria, Prevotella, and Desulfobivrio*	-	ASD	18 children	2 years follow up	↑Relative abundance of Bifidobacteria and *Prevotella* during 2 years follow up while ↓ relative abundance of *Desulfobivrio* in the last 18 weeks	[234]

**Note:** This table represents a list of probiotics in humans with ASD in clinical study (↑: Increase,↓: Decrease,+: Stimulate, –: Inhibited). **Abbreviations:** RCT: Randomized clinical trial, IBS: Irritable bowel syndrome, OCD: Obsessive-compulsive disorder, CGI: Clinical global impression.

## LIST OF ABBREVIATIONS

ENS: Enteric Nervous System
CNS: Central Nervous System
ASD: Autism Spectrum Disorder
DSM-V: Diagnostic and Statistical Manual of Mental Disorders-V
CDC: Community
5-HT: Serotonin
SHANK: SH3 Multiple Ankyrin Repeat Domain
CNTNAP: Contactin Association Protein
NLGN: Neuroligin
NRXN: Neurexin
CNV: Copy Number Variation
UBE3A: Ubiquitin Protein Ligase 3A
FMR1: Fragile X Messenger Ribonucleoprotein 1
TSC1: Tuberous Sclerosis 1
MECP2: Methyl CpG Binding Protein 2
FMRP: Fragile X Mental Retardation Protein
GLT1: Glutamate Transporter-1
PTEN: Phosphatase Tensin Homolog
BDNF: Brain-derived Neurotrophic fActor
LTP: Long-term Potentiation
LTD: Long-term Depression
AMPA: α-amino-3 Hydroxy-5 methyl-4-isoxazolepropionic Acid
NMDA: N-methyl-D- Aspartate
GABA: Glutamate Amino Butyric Acid
NMDA: N-methyl-D-Aspartate
BBB: Blood Brain Barrier
IL-17: Interleukin-17
CRH: Corticotropin-releasing Hormone
CRF: Corticotropin Factor
HPA: Hypothalamus Pituitary Axis
SCFA: Short-chain Fatty Acid
CNS: Central Nervous System
ADHD: Attention Deficit Hyperactivity Disorder
MIA: Maternal Immune Activation
APCs: Antigen-presenting Cells
GFCP: Gluten and Casein-free Diet
DNA: Deoxy Ribonucleic Acid
FMRI: Functional Magnetic Resonance Imaging
BOLD: Blood Oxygen Level-dependent
PHMRI: Physiological Magnetic Resonance Imaging
GI: Gastro Intestinal
MGS: Metagenomic Sequencing
IBD: Inflammatory Bowel Diseases
Iga: Immunoglobulin A
HMP: Human Microbiome Project
GBA: Gut-brain axis
TNF: Tumor Necrosis Factor
IFN Gamma: Interferon Gamma
LEARN: Latency Early Life Regulation
MCP-1: Monocyte Chemoattractant Protein-1
ROS: Reactive Oxygen Species
RNS: Reactive Nitrogen Species
NAD: Nicotinamide Adenine Dinucleotide
FAD: Flavin Adenine Dinucleotide
NADH: Nicotinamide Adenine Dinucleotide + Hydrogen(H)
FADH2: Flavin Adenine Dinucleotide Hydride 2
ETC: Electron Transport Chain
PPA: Propionic Acid
MAM: Mitochondrial Association Membrane
GTPase: Guanosine Triphosphatase
CSF: Cerebrospinal Fluid
APA: American Psychiatric Association
MRI: Multiple Resonance Imaging
WHO: World Health Organisation
MD: Mediterranian Diet
SCD: Specific Carbohydrate Diet
GF: Gluten-free
CFD: Casein-free Diet
PUFA: Poly Unsaturated Fats
FMT: Fecal Microbiota Transplantation
GSRS: Gastrointestinal Symptom Rating Scale
CARS: Childhood Autism Rating Scale
SRS: Social Responsiveness Scale
ABC: Aberrant Behaviour Checklist
MDM|A: Methylenedioxymethamphetamine
SAD: Social Anxiety Disorder
FDA: Food Drug Administration
BGM: Brain Gut Microbiota
CFU: Colony Forming Unit
MS: Multiple Sclerosis
PD: Parkinson's Disease
MLNs: Mesenteric Lymph Nodes
